# Baseline audiological profiling of South African females with cervical cancer: an important attribute for assessing cisplatin-associated ototoxicity

**DOI:** 10.1186/s12905-021-01313-5

**Published:** 2021-04-20

**Authors:** Jessica Paken, Cyril D. Govender, Mershen Pillay, Birhanu T. Ayele, Vikash Sewram

**Affiliations:** 1 Discipline of Audiology, School of Health Sciences, University of KwaZulu-Natal, Private Bag X54001, Durban, 4000 South Africa; 2 Division of Epidemiology and Biostatistics, Stellenbosch University, P.O. Box 241, Cape Town, 8000 South Africa; 3 Department of Global Health, African Cancer Institute, Faculty of Medicine and Health Sciences, Stellenbosch University, P.O. Box 241, Cape Town, 8000 South Africa

**Keywords:** Cisplatin ototoxicity, Cervical cancer, Diabetes, Hearing, HIV, Hypertension, Ototoxic medication, Risk factors

## Abstract

**Background:**

Cisplatin is a popular antineoplastic agent used to treat cervical cancer in women from low and middle-income countries. Cisplatin treatment is associated with ototoxicity, often resulting in hearing loss. In light of this, it is crucial to conduct baseline audiological assessments prior to treatment initiation in order to evaluate the extent of cisplatin-associated-ototoxicity. Additionally, the identification of inherent risk factors and hearing patterns in specific patient cohorts is needed, especially in South Africa, a middle-income country characterized by the quadruple burden of disease (Human Immunodeficiency Virus (HIV), Tuberculosis (TB), Diabetes and Hypertension).

**Methods:**

This study aimed to describe a profile of risk factors and hearing in a cohort of females with cervical cancer before cisplatin treatment commenced. A descriptive study design that included 82 cervical cancer patients, who underwent audiological evaluation prescribed for ototoxicity monitoring was conducted.

**Results:**

All participants (n = 82) presented with risk factors (diabetes, hypertension, HIV, and antiretroviral therapy) for cisplatin ototoxicity and/or pre-existing sensorineural hearing loss. High-frequency tinnitus was the most common otological symptom experienced by 25 (31%) participants. Fifty-nine (72%) participants presented with normal hearing, twenty-two (27%) with a sensorineural hearing loss, and 36% were diagnosed with mild hearing loss. Abnormal Distortion Product Otoacoustic Emissions (DPOAE) findings were obtained bilaterally in two participants (2.4%), in the right ear only of another two (2.4%) participants and the left ear of three participants (3.7%). Most participants (94%) had excellent word recognition scores, demonstrating an excellent ability to recognize words within normal conversational levels under optimal listening conditions. Age was significantly associated with hearing loss at all thresholds. Among the co-morbidities, an HIV positive status significantly triggered hearing loss, especially at higher frequencies.

**Conclusion:**

This study demonstrated that South African females with cervical cancer present with various co-morbidities, which may predispose them to develop cisplatin-associated -ototoxic hearing loss. Identification of these co-morbidities and hearing loss is essential for the accurate monitoring of cisplatin toxicities. Appropriate management of these patients is pivotal to reduce the adverse effects that hearing impairment can have on an individual’s quality of life and to facilitate informed decision-making regarding the commencement of cisplatin chemotherapy.

**Supplementary Information:**

The online version contains supplementary material available at 10.1186/s12905-021-01313-5.

## Background

Hearing loss has now been identified as the fourth highest cause of disability globally, with South Asian, Asia Pacific, and Sub-Saharan African regions being the most affected. This is evident by prevalence rates being almost four times higher in low-income compared to high-income regions [1]. While the aging population is one of the main contributors to this high prevalence, others include noise exposure (occupational and recreational), chronic ear infections, and ototoxicity [1]. With hearing loss being on the increase globally, the role of the audiologist in the early identification, diagnosis, and management of this “invisible condition” cannot be overstated. In an attempt to provide the best possible management for hearing loss, audiologists must have a clear understanding of audiological patterns affecting specific patient populations, through audiological profiling. As both occupational noise exposure and ototoxicity often result in progressive, permanent hearing loss, audiological monitoring for early identification is essential to audiological service delivery [1]. In any audiological monitoring program, the purpose of the baseline audiological assessment is to document the individual's hearing status before exposure to the noxious agent (either ototoxic drug or noise) [2]. While the effects of noise exposure are reduced or avoidable through the use of hearing protection devices, ototoxicity, on the other hand, is unavoidable. Ototoxicity results from exposure to ototoxic drugs, which are often the drug treatments used to treat various conditions such as Human Immunodeficiency Virus (HIV), tuberculosis (TB), and cancer [3].

Females with cervical cancer are identified as an “at-risk” cohort for ototoxicity in South Africa. It is the second most common cancer diagnosed in women in South Africa, with an age-standardized incidence rate of 26.96 per 100,000. Additionally, 1 in 35 women presents with a lifetime risk (0–74 years) of developing cervical cancer [4] coupled with late stage of diagnosis [5], resulting in treatments mainly confined to brachytherapy and cisplatin-containing-chemotherapy [6].

Cisplatin is a popular and effective antineoplastic drug used in the treatment of many cancer types, including cervical cancer; however, it is well-known for its ototoxic side effects [7]. The latter is due to the structures of the inner ear being susceptible to damage, and the outer hair cells in the basal turn of the cochlea being most affected [8]. The manifestation of cisplatin-induced-ototoxicity is high-frequency, progressive sensorineural hearing loss [9], which is often accompanied by tinnitus [10].

Further complicating the situation for women with cervical cancer is their diagnosis of HIV, which requires the concomitant use of antiretroviral therapy (ARTs). This is problematic since cervical cancer is regarded as an AIDS-defining illness, and HIV in itself, has been reported to cause hearing loss [11]. Proposed underlying mechanisms include the direct action of the virus on the central nervous system, including the 8^th^ nerve, or opportunistic infections associated with hearing loss [11]. Furthermore, ARTs are also reported to negatively affect hearing [12]. As a result, patients who are HIV positive and also have cervical cancer are a high-risk cohort for hearing loss, as there is likely to be an additive adverse effect on their hearing. Evidence shows that age [13, 14], cumulative dose [15, 16]¸ exposure to concomitant noise [17], chemicals and other ototoxic medications [16], as well as pre-exposure hearing ability [16], are known risk factors which may increase the severity of cisplatin ototoxicity.

Further complicating this disease profile is the increasing incidence of non-communicable diseases (NCDs) [18], including type 2 diabetes and hypertension [19], which are more prevalent than the country's BRICS counterparts, i.e., Brazil, Russia, India, and China [20]. Therefore, there is an increased likelihood of cervical cancer patients presenting with other co-morbidities, which may also have an impact on hearing abilities, as individuals with diabetes[21] and hypertension [22] have also been found to present with reduced hearing sensitivity at all frequencies.

Given the potential for combined co-morbidities, and the resultant exposure to ototoxic medication, the need for baseline audiological monitoring is pivotal to provide a frame of reference for future audiometric testing in complex disease profiles. Determining changes in hearing ability over time will significantly contribute to a better understanding of the associated risk characteristics as well as identify how hearing loss manifests in complex disease profiles before cisplatin chemotherapy. Furthermore, obtaining baseline audiometric results may prevent misinterpretation of high-frequency hearing loss when monitoring cisplatin ototoxicity, since presbycusis (hearing loss due to age) may mimic the configuration of cisplatin ototoxicity, as both manifest in a high-frequency sensorineural hearing loss [23]. Additionally, knowledge of individual risk and audiological profiles before treatment allows for accurate audiological monitoring of the effect of cisplatin. However, there needs to be due consideration of co-morbid conditions and confounding variables, of which many studies [15, 24–27] investigating hearing loss, to date, have failed to consider. This paper reports on the baseline audiological characteristics amongst patients with cervical cancer and reports on associated risk factors and confounding variables. Furthermore, we provide recommendations to implement prospective follow up visits to identify cisplatin-associated hearing loss.

## Methods

### Study design

Findings reported in this paper form part of a prospective cohort study among cervical cancer patients before cisplatin exposure.

### Setting

The study was conducted at a referral hospital offering tertiary services in KwaZulu-Natal (KZN), South Africa, as defined in the regulations relating to categories of hospitals [28]. This site was selected as it provides regional services to an approximate population of 1 million and tertiary services (highly specialized health care) to the Western half of KZN, i.e., five health districts with a total population of 3.5 million. It is also one of the main referral centers for cancer patients and houses an audiology department.

### Study sample

Patients attending the hospital’s oncology clinic who met all the inclusion criteria were identified, informed of the study and subsequently invited to participate by the clinicians, nurses as well as the primary investigator. Of the 86 patients who responded to the invitation, 82 adult females (≥ 18 years) with a diagnosis of cervical cancer were recruited before the commencement of the first cycle of cisplatin-based chemotherapy. Patients presenting with profound hearing loss at baseline assessment, or those who had previously received cisplatin chemotherapy or had a history of medical conditions such as tuberculosis, and malaria were excluded. Four of the 86 patients were excluded, as one had previously received chemotherapy, while the other three were treated with aminoglycoside antibiotics following a diagnosis of multi-drug resistant tuberculosis (MDR TB). All participants were tested for HIV, and those who were diagnosed as being HIV positive received antiretroviral therapy as part of the clinical management.

### Data collection

Following written informed consent, participants’ medical records were reviewed. Additionally, a structured questionnaire (Additional file 1) was used to solicit information on self-reported symptoms indicative of hearing loss, hearing history, medical history, family history of hearing loss, and history of noise exposure[29]. Audiological assessments were conducted on each participant following the review of the medical file. These assessments included otoscopy, tympanometry, ipsilateral and contralateral acoustic reflex threshold testing, pure tone air and bone conduction audiometry, extended high-frequency audiometry, speech reception threshold (SRT) testing, word recognition score (WRS) testing and distortion product otoacoustic emission (DPOAE) testing, consistent with the ototoxicity monitoring protocol, as prescribed by American Speech-Language-Hearing Association (ASHA) (1994) [2], and Health Professions Council of South Africa (HPCSA) (2018) [30]. A table reflecting the audiological procedures, motivation for its use, and the equipment utilized is presented in Additional file 2.

### Data analysis

Data is described using frequencies, percentages, medians, and ranges. The distribution of participant’s pure-tone air conduction thresholds (PTACT) at the different frequencies was analyzed separately for the left and right ear. Risk factor information was gathered from patients’ self-reports and medical records and was stratified into two risk categories (low-risk category ≤ two risk factors; high-risk ≥ three risk factors). The results of the audiological assessment were analyzed as per normative data indicated in Additional file 3.

The Tobit (censored) regression was used to estimate the linear relationship between hearing loss and risk factors, adjusted for age, as it accounted for non-responses (values above the limits of the audiometer, as indicated in Additional file 4) at the various pure tone frequencies. Methods that consider non-responses as actual values usually bias the estimate of the coefficients leading to incorrect conclusions. All statistical analyses were conducted using SAS 9.4 (Johannesburg, SA).

### Reliability and validity

The reliability of results was ensured by using all standard audiological tests and procedures to ensure consistency. Case history ascertainment was confirmed by reviewing medical records. The cross-check principle was employed during audiological evaluations. All equipment was calibrated by a qualified technician annually, in accordance with the South African National Standards set by the South African Bureau of Standards (SABS) with daily biological checks conducted by the primary researcher.

## Results

### Descriptive analysis

Data from 82 female patients with cervical cancer were analyzed, and the demographic and medical characteristics are summarized in Table 1. The median age of the cohort was 52 years (range 32–79 years). Furthermore, 37 (45.1%) participants presented with stage IIB, 29 (35.4%) presented with stage IIIB cervical cancer, whereas stage IA, IB, and IIIA were less common (less than 4% each). Sixty-eight participants (82.9%) presented with co-morbidities, of which 44 (64.7%) were HIV positive and on ARTs. The 5 patients with stage 1 cancer received chemotherapy either due to peri-neural involvement on histology, lymph node involvement post-surgery, whilst another developed a local recurrence post-hysterectomy.

**Table 1:** Demographic and medical characteristics of the participants (N = 80)

Category	Number (%)
*Age*	
< = 39	11 (13.4)
40–49	25 (30.5)
50–59	25 (30.5)
> = 60	21 (25.6)
Total	82
*Ethnic group*	
Black African	75 (91.5)
Indian/Asian	4 (4.9)
Coloured	3(3.6)
Total	82
*Stage of cancer*	
I A	3 (3.7)
I B	2 (2.4)
II A	9 (10.9)
II B	37 (45.1)
III A	2 (2.4)
III B	29 (35.4)
Total	82
*HIV status*	
Positive	44 (53.7)
Negative	38 (46.3)

A summary of the self-reported audiological symptoms is presented in Table 2. Firstly, nine (11.0%) participants reported reduced hearing sensitivity, of which five (55.6%) were bilateral. Tinnitus was the most common self-reported symptom experienced by 28 participants (34.0%), with 25 of these participants (89.3%) describing the tinnitus as high-frequency in nature. Only two participants (2.4%) reported repeated ear infections.

**Table 2:** Self- reported audiological symptoms at baseline

Symptoms	n (%)
Reduced hearing sensitivity	9 (11%)
Ear associated with reduced hearing sensitivity (n = 9)	Both-5 (55.6)
	Left only-3 (33.3)
	Right only-1(11.1)
Otalgia	5 (6)
Aural fullness	4 (4.9)
Tinnitus	28 (34)
Ear associated with tinnitus	Both-15 (54)
	Left only-10 (35)
	Right only-1 (4)
	Head-2 (7)
Description of tinnitus (n = 28)	High frequency-25 (89.3)
	Low frequency-2 (7.1)
	Pulsating-1 (3.6)
Repeated middle ear infections	2 (2.4)

Abnormal otoscopic findings (in this case, tympanic membrane perforation) were evident in the right ear of one participant (1.0%), and the left ear of another participant (1.0%). Tympanometric findings revealed normal results, i.e., Type A tympanograms in 81 (98.8%) participants bilaterally. Acoustic reflex threshold testing revealed abnormalities in the right ear of 33 participants (40.0%) and the left ear of 38 participants (46.0%).

Moreover, hearing loss was identified in the right ear of five participants (6.0%) in the left ear of six participants (7.0%) and bilaterally in 17 participants (21.0%). Of the participants with bilateral hearing loss, five (17.0%) had indicated reduced hearing sensitivity. Among those participants with bilateral hearing loss (n = 17), five (17.0%) reported experiencing tinnitus. Complaints of tinnitus were also reported by two (33.3%) of the six participants with hearing loss in the left ear only and two (28.7%) of the seven participants with hearing loss in the right ear only. Mixed hearing loss was identified in the right ear of one participant and the left ear of another, with the remaining participants presenting with sensorineural hearing loss. Mild hearing loss was most common (35.0%), followed closely by mild-moderate, and this pattern was evident bilaterally.

**Fig. 1:**
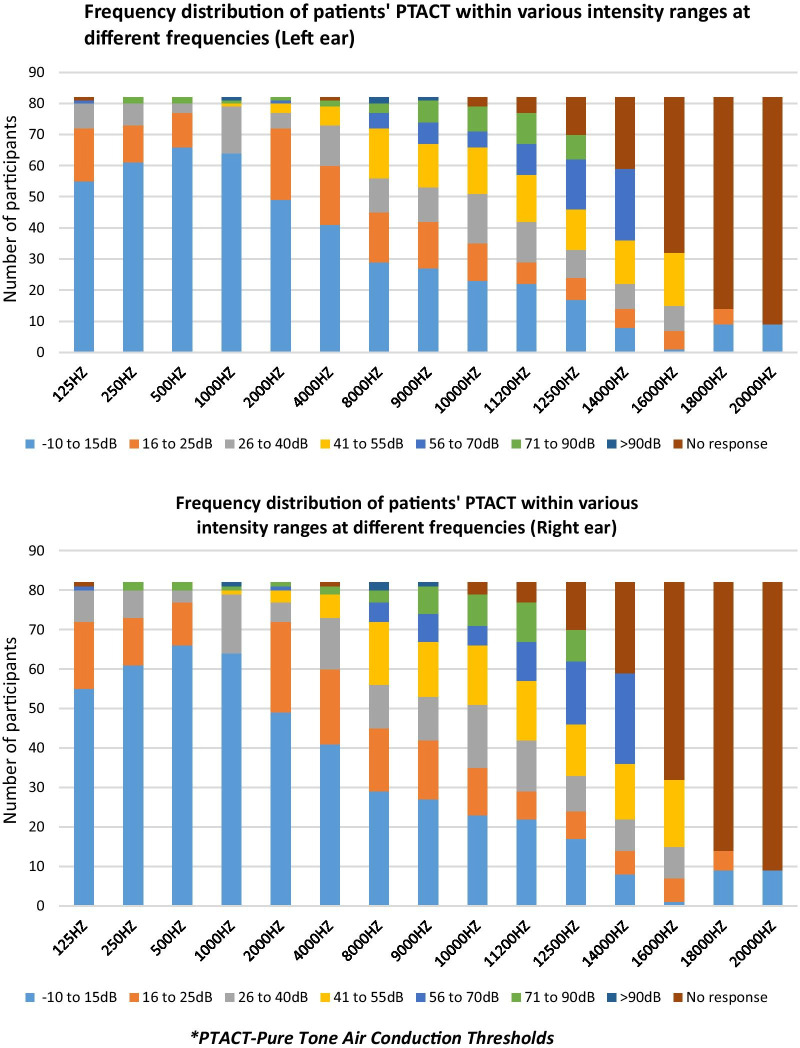
Percent distribution of participants’ PTACT within the various intensity ranges at the different frequencies for the left and right ear

A steady decline in the number of participants with hearing thresholds between -10 and 25 dB was seen as the frequency increased, as indicated in Fig. 1. Conversely, the number of participants with no responses steadily increased as the frequency increased. Abnormal DPOAE findings were obtained bilaterally in two participants (2.4%), in the right ear only of another two (2.4%) participants and the left ear only of another three participants (3.7%).

Additionally, the speech reception threshold (SRT) testing revealed good SRT-PTA correlation in more than 95% of the participants bilaterally; thus, confirming the validity of pure-tone test results. Word recognition score testing showed that most participants had an excellent ability to understand speech within normal conversational levels under optimal listening conditions.

Moreover, 73 (89%) participants presented with one or more risk factors for hearing loss, as indicated in Table 3. Fourteen of the 18 participants (77.8%), categorized as high risk, were HIV positive.

**Table 3:** Overview of the study population

No. of risk factors	Risk factors					
	Patients number (N = 82)	Diabetes (n = 10)	Hypertension (n = 28)	HIV infection (n = 44)	Ototoxic medication (n = 45)	Pre-existing hearing loss (n = 28)
0	9	0	0	0	0	0
1	12	2	5	0	0	5
2	43	2	13	30	31	10
3	16	4	8	12	12	12
4	1	1	1	1	1	0
5	1	1	1	1	1	1

Additionally, simple and multiple Tobit regressions were fitted to test the association between the hearing loss at various frequencies and covariates such as age, HIV status, NCDs (hypertension, diabetes co-morbidities), and use of ototoxic medication. All HIV positive patients took ototoxic medication, supporting a significant correlation (*p* < 0.0001) between HIV status and the use of ototoxic medication. Hence, we excluded the use of ototoxic medication to avoid the multi-collinearity problem. Age, being a confounding variable, was accounted for in the multiple regression model. Results of simple and multiple regression models are depicted in Tables 4 and 5 for the right ear and Tables 6 and 7 for the left ear, respectively. In the simple Tobit regression, age was found to be a significant predictor of thresholds at all (low to extended high) frequencies (Table 4, 6). The direction of the association is positive, indicating that the older the patient, the higher the risk of hearing loss.

**Table 4:** Simple Tobit regression (right ear)

Frequency (Hz)	No. of non-responses	Risk factors Estimate of coefficient (SE); *p*-value		
		Age	HIV Seropositive	NCD
*LFPTT*				
125	0	0.2 (0.09); 0.02	1.15 (1.98); 0.56	2.33 (2.16); 0.28
250	0	0.26 (0.1); 0.01	3.8 (2.24); 0.09	− 0.42 (2.58); 0.87
500	0	0.25 (0.1); 0.01	5.89 (2.23); 0.01	− 0.46 (2.66); 0.86
*MFPTT*				
1000	1	0.11 (0.12); 0.38	3.52 (2.68); 0.19	− 2.52 (3.08); 0.41
2000	0	0.32 (0.13); 0.01	3.33 (3.04); 0.27	− 0.3 (3.54); 0.93
*HFPTT*				
4000	0	0.56 (0.14); 0.01	8.21 (3.2); 0.01	1.0 (3.71); 0.79
8000	0	0.86 (0.17); 0.01	10.97 (4.02); 0.01	3.21 (4.6); 0.48
9000	0	1.13 (0.17); 0.01	11.4 (4.51); 0.01	4.37 (5.17); 0.39
*EHFPTT*				
10,000	1	1.29 (0.19); 0.01	14.41 (4.97); 0.01	2.64 (5.83); 0.65
11,200	1	1.37 (0.20); 0.01	16.90 (5.25); 0.01	3 (6.17); 0.63
12,500	12 (14.6%)	1.79 (0.21); 0.01	21.57 (5.78); 0.01	− 5.45 (6.92); 0.43
14,000	31 (37.8%)	2.04 (0.27); 0.01	24.62 (6.63); 0.01	− 3.95 (7.88); 0.62
16,000	57 (70%)	1.64 (0.34); 0.01	22.16 (7.06); 0.01	− 5.1 (6.85); 0.46
18,000	75 (91%)	0.47 (0.22); 0.03	5.31 (3.84); 0.17	1.06 (2.92); 0.72
20,000	79 (96%)	0.42 (0.51); 0.14	8.7 (0.88); 0.01	− 0.95 (1.23); 0.44

A positive coefficient of an estimate indicates that the mean of hearing loss increase with the value of the potential risk factor. The higher the estimate the higher the hearing loss

Reference categories: HIV = 0 and NCD (Hypertension and Diabetes) = 0

LFPTT, Low-frequency pure tone thresholds; MFPTT, Mid-frequency pure tone thresholds, HFPTT, High frequency pure tone thresholds; EHFPTT Extended high-frequency pure tone thresholds

**Table 5:** Multiple Tobit regression adjusting for age (right ear)

Frequency (Hz)	No. of non-responses	Risk factors Estimate of coefficient (SE); *p*-value	
		HIV seropositive	NCD
*LFPTT*			
125	0	0.79 (2.78); 0.77	2.42 (2.13); 0.26
250	0	1.72 (3.26); 0.59	− 0.01 (2.50); 0.99
500	0	4.17 (4.02); 0.3	0.26 (2.56); 0.92
*MFPTT*			
1000	1	3.52 (2.68); 0.19	− 2.52 (3.08); 0.41
2000	0	1.81 (4.52); 0.69	− 0.17 (3.46); 0.96
*HFPTT*			
4000	0	1.93 (3.2); 0.67	1.70 (3.42); 0.62
8000	0	1.07 (5.15); 0.83	3.93 (3.94); 0.32
9000	0	4.18 (5.32); 0.43	5.06 (4.07); 0.21
*EHFPTT*			
10,000	1	5.51 (5.84); 0.34	3.32 (4.47); 0.46
11,200	1	4.40 (6.21); 0.01	3.88 (4.76); 0.41
12,500	12 (14.6%)	8.32 (6.02); 0.16	− 5.96 (4.54); 0.38
14,000	31 (37.8%)	6.42 (6.79); 0.34	− 0.49 (5.33); 0.93
16,000	57 (70%)	0.99 (7.71); 0.89	0.76 (6.0); 0.89
18,000	75 (91%)	0.03 (3.45); 0.99	3.53 (2.99); 0.24
20,000	79 (96%)	9.58 (4.08); 0.02	− 0.26 (0.48); 0.59

LFPTT, Low-frequency pure tone thresholds; MFPTT, Mid-frequency pure tone thresholds; HFPTT, High frequency pure tone thresholds; EHFPTT, Extended high-frequency pure tone thresholds; NCD, Hypertension and Diabetes

**Table 6:** Simple Tobit regression (left ear)

Frequency (Hz)	No. of non-responses	Variables Estimate of coefficient (SE); *p*-value		
		Age	HIV seropositive	NCD
*LFPTT*				
125	1	0.36 (0.12); 0.01	0.95 (2.77); 0.73	3.51 (3.14 (0.26)
250	0	0.41 (0.13); 0.01	0.71 (3.04); 0.82	3.5 (3.42); 0.31
500	0	0.4 (0.14); 0.01	0.36 (3.28); 0.91	4 (3.76); 0.28
*MFPTT*				
1000	0	0.42 (0.16); 0.01	0.88 (3.56); 0.8	3.21 (4.02); 0.42
2000	0	0.55 (0.16); 0.01	2.21 (3.66); 0.55	3.25 (4.11); 0.43
*HFPTT*				
4000	1	0.91 (0.17); 0.01	7.14 (4.29); 0.09	7.26 (4.89); 0.14
8000	0	1.11 (0.2); 0.01	9.77 (5.07); 0.05	1.62 (5.77); 0.01
9000	1	1.33 (0.19); 0.01	16.11 (4.92); 0.001	4.13 (5.82); 0.48
*EHFPTT*				
10,000	3	1.64 (0.19); 0.01	21.23 (5.28); 0.01	5.25 (6.58); 0.42
11,200	5	1.95 (0.19); 0.01	25.3 (5.61); 0.01	0.54 (7.15); 0.94
12,500	12 (14.6%)	2.02 (0.19); 0.01	26.4 (5.76); 0.01	− 1.81 (7.36); 0.81
14,000	23 (28%)	1.85 (0.21); 0.01	22.37 (5.76); 0.01	4.38 (7.16); 0.54
16,000	50 (61%)	0.92 (0.23); 0.01	12.93 (4.81); 0.01	6.87 (5.67); 0.22
18,000	68 (83%)	0.71 (0.46); 0.13	6.1 (9.42); 0.52	11.85 (9.62); 0.22
20,000	73 (89%)	0.23 (0.21); 0.26	1.81 (4.34);0.68	4.74 (4.99);0.34

LFPTT, Low frequency pure tone thresholds; MFPTT, Mid frequency pure tone thresholds; HFPTT, High frequency pure tone thresholds; EHFPTT, Extended high frequency pure tone thresholds; NCD, Hypertension and Diabetes

**Table 7:** Multiple Tobit regression adjusting for age and co-morbidities (left ear)

Frequency (Hz)	No. of non-responses	Risk factors Estimate of coefficient (SE); *p*-value	
		HIV seropositive	NCD
*LFPTT*			
125	1	11.59 (3.69); 0.01	2.72 (2.81); 0.33
250	0	9.59 (4.11); 0.02	2.93 (3.14); 0.35
500	0	9.66 (4.6); 0.04	3.42 (3.52); 0.33
*MFPTT*			
1000	0	6.74 (5.05); 0.18	2.91 (3.86); 0.45
2000	0	7.65 (4.98); 0.12	2.99 (3.81); 0.43
*HFPTT*			
4000	1	9.68 (5.36); 0.07	7.19 (4.09); 0.08
8000	0	6.5 (6.37); 0.31	2.03 (4.87); 0.68
9000	1	1.02 (5.93); 0.86	5.29 (4.51); 0.24
*EHFPTT*			
10,000	3	0.07 (6.18); 0.99	6.73 (4.68); 0.15
11,200	5	0.90 (6.0); 0.88	2.11 (4.55); 0.64
12,500	12 (14.6%)	2.87 (5.84)0.62	− 0.68 (4.42); 0.88
14,000	23 (28%)	8.25 (6.21); 0.18	6.11 (4.72); 0.19
16,000	50 (61%)	− 1.3 (6.76); 0.85	11.35 (5.54); 0.04
18,000	68 (83%)	5.68 (12.71); 0.65	15.23 (10.54); 0.15
20,000	73 (89%)	1.75 (5.92); 0.77	5.56 (5.16); 0.28

LFPTT, Low-frequency pure tone thresholds; MFPTT, Mid-frequency pure tone thresholds; HFPTT, High-frequency pure tone thresholds; EHFPTT, Extended high-frequency pure tone thresholds; NCD, Hypertension and Diabetes

Similarly, HIV status was predictive at high and extended high-frequency thresholds in the simple Tobit regression. The percentage of non-responses was very high at frequencies of 16 kHz, 18 kHz, and 20 kHz. This ranged from 70 to 96% for the right ear and from 61 to 89% for the left ear, respectively. Hence, we did not interpret results at these frequencies, as interpreting information obtained from a small sample is unreliable.

## Discussion

In this study, we report for the first time novel findings on baseline audiological profiles and risk factors, which may further exacerbate hearing loss in patients with cervical cancer, a cohort already at risk of reduced quality of life. Cervical cancer is the second most common cancer among females in the African continent, including South Africa [31], which is also burdened with a high prevalence of infectious and NCDs [20]. We report that 89% of the participants in the current study presented with co-morbid conditions (HIV, diabetes, and hypertension), as reflected by the risk profile. A multitude of risk factors can profoundly decrease the quality of life of these participants. Consequently, it becomes critically important that the medical and rehabilitation fraternity consider the patient’s quality of life, especially since more people with chronic conditions and compromised immune systems are living longer, due to greater access to treatments. In light of this, we need to acknowledge the risk profile of these cervical cancer patients receiving cisplatin and understand the depth to which hearing loss may impinge on this cohort by either prevention and/or treatment in order to avoid further increasing the risk for hearing loss and compromising the quality of life.

Acknowledging the risk profile may also result in a greater realization of the complexity of the synergistic effects of these risk factors for cisplatin ototoxicity. Simultaneous exposure to multiple risk factors (co-morbidities and ototoxic medication) can potentiate auditory dysfunction that is greater than the sum of each insult given individually, implying a synergistic effect [32]. In agreement, an in vivo model by Bielefeld et al. demonstrated an additive effect of cisplatin and increasing age on the cochlear [33]. Additionally, a study by Gratton et al. [17] showed that the combination of cisplatin and moderate-to-high levels of noise caused significantly more hair cell damage as well as hearing loss at the higher frequencies compared to either noise or cisplatin alone. While each of the changes may present clinically or audiologically differently, they often combine and result in a bilateral mild to moderate mid to high-frequency sensorineural hearing loss [34]. The effects of aging on hearing sensitivity could be worsened by the use of certain ARTs as a result of mitochondrial toxicity, which results in accelerated changes in the cochlear and/or central auditory system [35]. This may be plausible in the current study, as there is a high prevalence of HIV positive cases (53.7%) in this cohort.

Furthermore, if a person presents with more than one of these medical conditions, i.e., diabetes, hypertension, and/or HIV as they age, there is likely to be an even greater antagonistic, additive effect on hearing that may progressively worsen during the course of cisplatin chemotherapy. This, therefore, reinforces the importance of the audiologist conducting a detailed case history at baseline so that they may be able to identify probable reasons for the otological symptoms experienced as well as the audiological profile of the patient.

A review of the audiological profile at baseline revealed high-frequency tinnitus to be the most common self-reported otological symptom. While tinnitus is often associated with a hearing loss, the number of participants with self-reported hearing difficulties and even those with clinical hearing loss was lower than the complaints of tinnitus in our study. This finding should be of no surprise, taking into consideration that most participants presented with other medical conditions, namely diabetes, hypertension, and HIV, and its associated treatments of which has been reported to cause tinnitus as a side effect [3]. These findings are in agreement with Shargorodsky et al. [36], who reported the prevalence of tinnitus to be 25.3% in a sample of the general population in the United States of America. Furthermore, we report that seven of the participants with hearing loss also complained of tinnitus, an early indicator of auditory dysfunction [37].

Complaints of otalgia appeared to be much lower than expected (6%) when compared to previous literature focusing on HIV infected participants in South Africa, which indicated that 19% of the sample reported otalgia [11]. It is, however, in agreement with the findings of Tuz et al., who reported that otalgia was experienced by 8% of their control group participants [38]. Otalgia may be a result of pain within the ear or ‘referred pain,’ i.e., due to pain in structures around the ear or other head and neck structures [39]. With only two patients reporting a history of ear infections and two displaying abnormalities on otoscopic examination and tympanometry, the otalgia could likely be attributed to these clinical findings.

The small number of participants presenting with a history of ear infections and/or otoscopic and tympanometric abnormalities is a rare contradictory finding because more than 50% of the current study participants are HIV positive and previous reports otitis media to be most common in this population [40, 41]. However, this finding could be attributed to the fact that all participants diagnosed with HIV were receiving ARTs. While the duration of treatment with ARTs is unknown, it can be speculated that it is longer than a period of six months as patients generally commenced with treatment no earlier than six months after being diagnosed with cervical cancer, which would have prompted an HIV diagnostic test and the subsequent treatment with ARTs. The use of ARTs has proven to significantly improve the functioning of the immune system of HIV infected individuals, which indirectly results in less frequent middle ear abnormalities such as otitis media [11].

The percentage of participants with hearing loss (27%) at baseline in this current study is in agreement with Nagy et al., who reported that 26% of the study sample presented with hearing abnormalities [23]. The number of complaints of difficulty hearing was much lower compared to the audiological assessment, indicating that participants may have gradually adjusted to the reduced hearing sensitivity due to the loss being gradual in nature. This is generally seen in presbycusis and is consistent with the age characteristics of our study population. Additionally, literature reports that hearing loss is usually only suspected or detected when communication difficulties become evident [42], and may go unnoticed in the case of mild hearing loss, as evident in the current study, with mild hearing loss being the most common bilaterally.

Furthermore, if the extended high-frequency audiometry thresholds were considered in the general classification of hearing loss, more participants would likely be presenting with hearing loss. However, due to the lack of consensus around normative data for the extended frequency range, none of the classification systems for hearing loss consider this frequency range. Consequently, this data was merely used in this baseline study to reflect that the audiometric patterns were of a sloping configuration. The sloping configuration of the audiological patterns is congruent with other test findings and the demographic and medical profile of the participants. These findings are in agreement with previous reports, as with increasing age [43], hypertension [22], diabetes [44], HIV [41], and the use of ototoxic medication [3], a high-frequency sensorineural hearing loss is initially evident.

The highly limited occurrence of conductive hearing loss is in agreement with other South African studies [11, 45, 46]. The higher percentage of individuals presenting with sensorineural hearing loss (96%) may be explained by the fact that more than 60% of participants were HIV positive currently being treated with ARTs, which are considered ototoxic [3]. Additionally, other co-morbidities, including diabetes and/or hypertension [22, 45, 47–49], as well as aging [43], are all well-known to affect cochlear functioning, which consequently results in sensorineural hearing loss. Hence, indicating that this cohort of participants is at a higher risk for permanent hearing loss.

Due to the sensorineural nature of the hearing loss, one would expect most of these participants to present with reduced or absent DPOAEs. DPOAEs are generally absent in frequency regions with pure tone thresholds greater than 50 dB [50]. Therefore, with most participants in the study presenting with normal hearing or mild degrees of hearing loss, it is likely that these individuals may have presented with DPOAE amplitudes greater than 6 dB at four or more of the DPOAE test frequencies, resulting in the DPOAE result being considered as normal, as is seen in the current study.

Most participants presenting with normal hearing or mild degrees of hearing loss may also account for the excellent word recognition scores. This, therefore, corroborates with the small number of participants self-reporting hearing difficulties. Our findings are in agreement with that of Sooy [51], who also reported word recognition scores above 82%, with the majority of participants obtaining scores above 90% despite presenting with abnormal audiological findings. It may, therefore, be necessary to include speech in noise tests during baseline evaluations to stress the auditory system by portraying a ‘real world’ scenario. While impaired word recognition scores were expected, the results of this assessment may have been influenced by the use of monitored live voice testing and speech tests that have not been standardized for isiZulu speaking individuals, which comprised more than 90% of the cohort. Despite the many disadvantages of monitored live voice testing [52], this method of presentation was utilized due to the lack of the necessary equipment at the study site, a common issue affecting many institutions in low and middle-income countries.

Furthermore, due to the absence of a validated speech wordlist for isiZulu speakers, a decision was taken to utilize the Digits test for speech recognition threshold testing [53] and a speech word list which is routinely used in KZN hospitals. As the validity of this speech word list has not been established, it is likely that this tool may not adequately stress the auditory system to allow for an accurate description of the individuals’ ability to recognize speech and should, therefore, be viewed with caution. However, despite word recognition scores not being severely compromised, and most participants not presenting with debilitating degrees of hearing loss at this stage, they would still require counseling. Audiologists should still counsel their patients about the effects of concomitant exposure to risk factors on hearing as well as the effects of cisplatin on hearing at the baseline assessment, to facilitate informed decision-making and a greater awareness of the side effects of cisplatin chemotherapy.

While the current study has been conducted in South Africa, other countries experience similar issues regarding disease complexity, as reflected by the World Health Organization report (2018). In 2014, the World Health Organization indicated that one in four men and one in five women (i.e., 22% of the adult population aged 18 years and older) had hypertension globally, while the number of people with diabetes has nearly quadrupled since 1980 from 108 to 422 million in 2014 [54]. Thus indicating that cervical cancer patients receiving cisplatin chemotherapy may experience an increased risk of hearing complications. This may be true since there is an increase in the prevalence of cancer patients presenting with other co-morbidities while receiving cisplatin treatment. These current study findings bear testimony to the development of appropriate treatment management protocols of cisplatin related toxicities, e.g., an ototoxicity monitoring programme, in order to improve overall quality of life in cancer patients.

## Conclusion

This study has demonstrated that this cohort of South African women with cervical cancer presented with various risk factors, such as HIV infection, diabetes, hypertension, ototoxic medication, and pre-existing hearing loss, all of which may predispose them to develop cisplatin hearing loss. Considering that South Africa is burdened with a high prevalence of both infectious and NCDs, it is essential that a patient’s hearing is assessed before commencing cisplatin chemotherapy. This will enable the identification of known risk factors in an attempt to manage the patient as well as accurately monitor the impact of cisplatin ototoxicity. Our findings revealed the presence of a clinical hearing loss in the absence of symptoms; mild high-frequency hearing loss may go unnoticed unless there is routine monitoring of patients before, during, and post-chemotherapy. Furthermore, permanent sensorineural hearing loss emphasizes the need for possible referrals to other healthcare professionals, including psychologists and occupational therapists as hearing loss may impact on all facets of life [55]. Additionally, there is an increased need to counsel the patient and significant others, such as partners, children, and friends to facilitate early implementation of communication enhancing strategies and reduce the adverse effects on quality of life associated with hearing impairment [55], especially in light of the diagnosis of cervical cancer.

## Supplementary Information

**Additional file 1**. Case History Questionnaire

**Additional file 2**. Audiological Equipment, Motivation, and Procedure

**Additional file 3**. Clinical Analysis of Data

**Additional file 4**. Limits of the audiometer

## Data Availability

The data that supports the findings of this study are available on request from the corresponding author.

## Abbreviations

ART: Antiretroviral therapy
DPOAE: Distortion product otoacoustic emission
HIV: Human Immunodeficiency Virus
KZN: KwaZulu-Natal
NCDs: Non-communicable diseases
SRT: Speech recognition threshold
WRS: Word recognition score/speech discrimination
TB: Tuberculosis
