# Supplementary material for: Baseline audiological profiling of South African females with cervical cancer: an important attribute for assessing cisplatin-associated ototoxicity
Source: BMC Womens Health. 2021 Apr 20;21:164. doi: 10.1186/s12905-021-01313-5 (PMC8056627; doi:10.1186/s12905-021-01313-5)
Supplement: Supplementary file 1 — Additional file 1. Case History Questionnaire [file 12905_2021_1313_MOESM1_ESM.pdf]

Baseline Audiological Profiling of South African Females with Cervical Cancer: An Important Attribute for Assessing Cisplatin-Associated Ototoxicity

[1]Jessica Paken, [1]Cyril D. Govender, [1]Mershen Pillay, [2]Birhanu T. Ayele, [2]Vikash Sewram

SUPPLEMENTARY FILE 1: CASE HISTORY QUESTIONNAIRE FOR PATIENTS IN ENGLISH

DATE: [ ]

1. PERSONAL INFORMATION

CODE [ ]

BIRTHDATE: [ ] AGE: [ ]

RACE [ ] GENDER: [ ]

AUDIOLOGICAL EVALUATION: BASELINE: [ ] CYCLE 4: [ ]

FOLLOW UP: 1 [ ] 3 [ ] 6 [ ]

2. HEARING HISTORY

2.1. Do you experience hearing difficulties? Yes No

2.2. When did you first notice the problem? [ ]

2.3. In which ear do you experience these difficulties? Left Right Both

2.4. Do you experience any pain in your ears? Yes No

2.5. Do you hear noises in your ears or head? Yes No

2.5.1. How long have you been hearing these noises?

	< 1 week	1 week
2 weeks	1 month	2 months
3 months	< 6 months	6 months
< 1 year	>1 year	

2.5.2. Where is the noise present?

Head	Left Ear	Right ear	Both ears

2.5.3. Describe the sound.

High pitched	Low pitched	Ringing	Pulsating	Roaring

2.5.4. When do you hear this sound?

Morning	Midday	Afternoon	Night
All the time			

2.5.5. Is it present?

Continuously	Intermittently

2.5.6. Describe the loudness of these noises

Not loud at all	Slightly loud	Moderately loud	Very loud

**2.5.7. Has these noises changed since you started the treatment

Yes	No

**2.5.8. Has the loudness of these noises changed?

Yes, louder now	Yes, quieter now	No

2.6. Do you ever have a feeling of fullness or stuffiness in your ears?

Yes	No

---

3. OTOLOGIC HISTORY

3.1. Have you ever had any repeated ear infections?

Yes	No

3.1.1. Which ear?

Right	Left	Both

3.2. Have you had any surgery on your ears?

Yes	No

3.2.1. If yes, please specify what surgery as well as the date. DATE

SURGERY [ ]

3.3.Have you ever had any injury to the head or neck region? Yes / No

3.4.If so, provide details [ ]

4. FAMILY HISTORY

4.1. Does anyone in your family have a hearing loss? Yes / No

4.2. If so, who? [ ]

4.3.What was the cause of the hearing loss? [ ]

5. GENERAL MEDICAL HISTORY (To be completed in conjunction with review of patients medical records)

5.1. Do you suffer from any other medical conditions? Yes / No

5.2. Name the conditions. [ ]

5.3. Have you ever been diagnosed with Tuberculosis (TB) and/or malaria? Yes / No

5.4. When were you diagnosed with cancer? [ ]

5.5. Did you receive any other treatment for the cancer? Yes / No

5.5.1. If so, what treatment did you receive?

Surgery	Radiation therapy	Both

MEDICAL REVIEW

5.6. Stage of cancer: ____

5.7. List all the medication and dosage.

Medication	Dosage

5.8. Renal function: ____

5.9. Current weight: ____

6. NOISE EXPOSURE HISTORY

6.1. Have you ever been exposed/or are exposed to loud noise for long periods of time?

Yes	No

6.2. If yes, please specify the type of noise? [ ]

** - denotes questions to be asked if the patient had experienced tinnitus prior to commencement of chemotherapy.
