# Supplementary material for: Baseline audiological profiling of South African females with cervical cancer: an important attribute for assessing cisplatin-associated ototoxicity
Source: BMC Womens Health. 2021 Apr 20;21:164. doi: 10.1186/s12905-021-01313-5 (PMC8056627; doi:10.1186/s12905-021-01313-5)
Supplement: Supplementary file 2 — Additional file 2. Audiological Equipment, Motivation, and Procedure [file 12905_2021_1313_MOESM2_ESM.pdf]

Baseline Audiological Profiling of South African Females with Cervical Cancer: An Important Attribute for Assessing Cisplatin-Associated Ototoxicity

[1]Jessica Paken, [1]Cyril D. Govender, [1]Mershen Pillay, [2]Birhanu T. Ayele, [2]Vikash Sewram

SUPPLEMENTARY FILE 2: AUDIOLOGICAL PROCEDURE, MOTIVATION AND EQUIPMENT

Audiological Procedure	Motivation	Equipment
1. Case history interview	• Allowed for accessing information on the presence of self-reported auditory and otologic symptoms such as tinnitus, otalgia and hearing difficulties as well as medical and family history of hearing loss.	Case History questionnaire
2. Otoscopic Examination	• Determine the status of the tympanic membrane and the external ear [1].	Agine otoscope
3. Tympanometry	• Evaluate the integrity of middle ear functionality [2].	GSI Tympstar V2 Impedance meter
4. Ipsilateral and contralateral acoustic reflex thresholds	• Provide information about the functioning of the middle ear and the sensory, neural, and motor pathways associated with the reflex arc [2].	GSI Tympstar V2 Impedance meter
5. Pure tone audiometry (air	• Determine the type and degree of hearing loss [2].	Conducted in a sound proof booth, using the twin channel Madsen Astera.

and bone conduction)	• Pure tone air conduction thresholds were obtained bilaterally at the following frequencies: 125, 250, 500, 1000, 2000, 4000, 8000, 9000, 10000, 11200, 12500, 14000, 16000, 18000 and 20000 Hz, while bone conduction thresholds were obtained bilaterally at 250, 500, 1000, 2000 and 4000Hz.	
6. Speech audiometry	• Speech reception threshold (SRT) testing was used for the confirmation of pure tone thresholds which, alerts the audiologist to invalid pure tone results [2]. Word recognition score (WRS) testing was conducted to measure how well the listener could understand speech as a function of the ability to differentiate sounds under optimum circumstances. The score is intended to be a measure of the clarity with which the patient hears speech [2].	Speech audiometry was conducted using live voice monitoring in a sound proof booth, using the Madsen Astera. Speech lists used include the Digits test for SRT and the CID W-22 Auditory test word list for English speaking participants, while an isiZulu wordlist collated in the Discipline of Audiology, was used for isiZulu speakers, during word recognition score testing.

7. Distortion Product Oto-acoustic emission	• OAEs are sensitive to hearing losses, resulting from outer hair cell damage. OAE results assist with the differentiation of cochlear vs. retro-cochlear disorders [3].	Maico Oto-acoustic emission

References

1. Martin F, Clark J: **Introduction to audiology**, 10th ed edn. Pearson; 2009.
2. Gelfand SA: **Essentials of Audiology**, Fourth edn. New York: Thieme Publishers; 2016.
3. Robinette MS, Glattke TJ: **Otoacoustic emissions**. In: *Audiology diagnosis.* Edited by Roeser RJ, Valente M, Hosford-Dunn H. New York: Thieme Medical Publishers; 2000: 503-526.
