# Supplementary material for: Baseline audiological profiling of South African females with cervical cancer: an important attribute for assessing cisplatin-associated ototoxicity
Source: BMC Womens Health. 2021 Apr 20;21:164. doi: 10.1186/s12905-021-01313-5 (PMC8056627; doi:10.1186/s12905-021-01313-5)
Supplement: Supplementary file 3 — Additional file 3. Clinical Analysis of Data [file 12905_2021_1313_MOESM3_ESM.pdf]

Baseline Audiological Profiling of South African Females with Cervical Cancer: An Important Attribute for Assessing Cisplatin-Associated Ototoxicity

[1]Jessica Paken, [1]Cyril D. Govender, [1]Mershen Pillay, [2]Birhanu T. Ayele, [2]Vikash Sewram

SUPPLEMENTARY FILE 3: CLINICAL ANALYSIS OF DATA

Audiometric results was analyzed according to the following guidelines indicated in literature.

1. Otoscopic Examination

Results were categorized as normal or abnormal:

An abnormal result would indicate that the different structures of the ear i.e. the pinna, ear canal, tympanic membrane and/or surrounding structures presents with abnormalities e.g.:

a) The pinna is abnormal and/or presenting with sores.

b) The ear canal is reddened, or presenting with sores, discharge, foreign bodies, excessive wax, impacted wax, or blood in the ear canal.

c) The tympanic membrane is retracted, perforated, or reddened.

d) There is pre auricular swelling and/or swollen mastoid.

2. Immittance Audiometry

2.1. Tympanometry

Tympanometry results were classified as normal or abnormal. Tympanometric tracings will be compared to Jerger's classification [1].

Type of tympanogram	Characteristics
Type A	All characteristics are within normal limits:

	• static compliance (0.2 – 2.0 ml), middle ear pressure (-100 to 100 daPa), ear canal volume (0.2 to 1.8 ml).<br>• seen in normal hearing individuals and those with sensorineural hearing loss.
As	• normal middle ear pressure and ear canal volume but low static compliance.<br>• seen in individuals with otosclerosis.
Ad	• normal middle ear pressure and ear canal volume but high static compliance.<br>• seen in normal hearing patients with highly flaccid tympanic membranes.
B	• Abnormal result where the ear canal volume is normal but there is no peak pressure.<br>• Seen in individuals with middle ear dysfunction.
C	• Abnormal result where the ear canal volume and static compliance is normal but peak pressure is less than -100daPa.<br>• Seen in patients with Eustachian tube dysfunction and retracted tympanic membranes.

2.2. Acoustic reflex threshold testing

Acoustic reflex thresholds were classified as normal based on normative data for the contralateral (70dBSL – 95dBSL as suggested by Metz, 1952) [2] and ipsilateral acoustic reflexes (3- 6 dB SL better than contralateral as suggested by Moller, 1962 & Fria et al., 1975) [3]. An abnormal result was obtained if the acoustic reflex thresholds were absent, reduced or elevated, based on the normative data.

3. Pure Tone Audiometry

The degree of hearing loss was determined using the Silman and Silverman's (1991) [4] magnitude of hearing impairment. In addition to these categories of hearing loss, the researcher included categories depicting the change in degree of hearing loss at frequency ranges namely mild-moderate, mild to moderately severe, mild to severe, mild to profound, moderate to moderately severe, moderate to severe, moderate to profound, moderately severe to severe, moderately severe to profound and severe to profound. Extended high frequency audiometry thresholds were not used to classify the degree of hearing loss, as these have not been considered in the various classification systems due to the lack of consensus around normative data for this frequency range.

The type of hearing loss was determine based on the comparison of the air and bone conduction threshold as follows: a) air and bone conduction thresholds within normal limits indicates normal peripheral hearing, b) air and bone conduction depressed in the absence of an air-bone-gap indicates sensorineural hearing loss, c) air and bone conduction depressed in the presence of an air-bone-gap indicates mixed hearing loss [5].

4. Speech audiometry

4.1. Speech recognition threshold

A comparison of the SRT and Pure tone average was conducted. If a value of 0-10dB was obtained, then the result for the pure tone assessment was considered valid [6].

4.2. Word recognition score

Word recognition scores were descriptively analysed using Hodgson (1980) [7], as a guideline and categorized into excellent, good, fair, poor, very poor and extremely poor [8].

5. Oto-acoustic emissions

DPOAE results were classified as normal or abnormal. An abnormal results was obtained when four of the six frequencies tested were found to be reduced or absent. Measurements were based on a difference between the DPOAE and the individual's noise floor in the frequency range of 500Hz and 8000Hz. An emission was considered present if the difference was equal to or greater than 6dB and the absolute amplitude greater than -10dB SPL [9].

References

1. Jerger J. Clinical experience with impedance audiometry. Arch Otolaryngol 1970, 92:311-324.
2. Feldman AS. Acoustic impedance-admittance battery. In Katz J, editor. Handbook of clinical audiology. 2nd ed. Baltimore: Williams and Wilkins Company; 1978. 356-374.
3. Northern JL, Grimes AM: Introduction to acoustic impedance. In Katz J, editor. Handbook of clinical audiology. 2nd ed. Baltimore: Williams and Wilkins Company; 1978. 344–355.
4. Silman S, Silverman CA. Auditory diagnosis: Prinicplies and applications. San Diego: Academic Press; 1991.
5. Martin F, Clark J. Introduction to audiology, 10th ed. Pearson; 2009.
6. Kramer SJ. Audiology: Science to practice. San Diego: Plural Publishing, Inc.; 2008.
7. Hodgson WR. Basic audiological evaluation. Baltimore: Williams and Wilkins; 1980.
8. Appana D, Joseph L, Paken J. An audiological profile of patients infected with multi-drug resistant tuberculosis at a district hospital in KwaZulu-Natal. The South African Journal of Communication Disorders 2016, 63(1):154.
9. Hall JW. Handbook of Otoacoustic Emissions. San Diego: Singular Publishing; 2000.
