# Supplementary material for: Baseline audiological profiling of South African females with cervical cancer: an important attribute for assessing cisplatin-associated ototoxicity
Source: BMC Womens Health. 2021 Apr 20;21:164. doi: 10.1186/s12905-021-01313-5 (PMC8056627; doi:10.1186/s12905-021-01313-5)
Supplement: Supplementary file 4 — Additional file 4. Limits of the audiometer [file 12905_2021_1313_MOESM4_ESM.pdf]

Baseline Audiological Profiling of South African Females with Cervical Cancer: An Important Attribute for Assessing Cisplatin-Associated Ototoxicity

[1]Jessica Paken, [1]Cyril D. Govender, [1]Mershen Pillay, [2]Birhanu T. Ayele, [2]Vikash Sewram

SUPPLEMENTARY FILE 4: LIMITS OF THE AUDIOMETER

FREQUENCIES (Hz)	INTENSITY (dB)	
	Right Ear	Left ear
**125**	80	75
**250**	100	100
**500**	115	115
**1000**	120	120
**2000**	115	115
**4000**	115	115
**8000**	100	100
**9000**	95	95
**10000**	90	90
**11200**	85	85
**12500**	75	75
**14000**	65	70
**16000**	50	50
**18000**	20	25
**20000**	0	0
